# Biosynthesis of silver nanoparticles using Onosma sericeum Willd. and evaluation of their catalytic properties and antibacterial and cytotoxic activity

**DOI:** 10.3906/kim-2007-1

**Published:** 2020-12-16

**Authors:** Selda DOĞAN ÇALHAN, Mustafa GÜNDOĞAN

**Affiliations:** 1 Department of Pharmaceutical Biotechnology, Faculty of Pharmacy, Mersin University, Mersin Turkey; 2 Department of Pharmaceutical Technology, Faculty of Pharmacy, Mersin University, Mersin Turkey

**Keywords:** *Onosma sericeum* Willd., green chemistry, silver nanoparticle, reduction, biological applications

## Abstract

In this study, silver nanoparticle (AgNP) synthesis was carried out using *Onosma sericeum* Willd. aqueous extract for the first time, with a simple, economical, and green method without the need for any other organic solvent or external reducing or stabilizing agent. A variety of AgNPs, all of different particle sizes, were synthesized by controlling the silver ion concentration, extract volume, temperature, and pH. It was determined that the optimum conditions for AgNP synthesis were 1 mM AgNO3, pH 8, 25 °C, 20 g/200 mL extract, silver nitrate, and extract ratio 5:1 (v/v). The AgNPs were defined using UV-Vis spectroscopy, field emission scanning electron microscopy (FESEM), energy dispersive X-ray analysis (EDAX), transmission electron microscopy (TEM), and X-ray diffraction (XRD). The particle size distribution and zeta potential measurements of the AgNPs were measured using the dynamic light scattering (DLS) technique. It was determined that the AgNPs with a particle size of less than 10 nm showed a higher catalytic effect in the reduction of 2-nitrobenzenamine. It was also found that these nanoparticles had a cytotoxic effect on the MCF-7 breast cancer cell line depending on dosage and time. The resulting IC50 values were between 76.63 µg/mL and 169.77 µg/mL. Furthermore, the biosynthesized AgNPs showed effective antibacterial activity against the *Acinetobacter baumannii* bacteria. The results of the study showed that synthesized AgNPs can have a promising role in biomedical and nanobiotechnology applications.

## 1. Introduction

Metal nanoparticles are used in various fields due to their low melting point, large surface area, and different electrical and chemical properties [1]. They are also notable for their promise in applications in areas such as hydrogen production [2], optical instruments [3], sensor design [4], and biomedical applications [5].

Metallic nanoparticles are used in many fields such as science, engineering, and medicine, and the synthesis of metallic nanoparticles produced using green methods from different sources with different properties is one of the most important topics for researchers [6].

Researchers continue to work extensively on silver nanoparticles (AgNPs) due to their antimicrobial and anticarcinogenic properties [7,8]. In addition to these biological properties, AgNPs have also attracted attention due to their catalytic and optical properties [9,10]. Because of these unique properties, AgNPs are used in the pharmaceutical, cosmetic, and textile industries [11–13]. AgNPs can be obtained through many different synthesis methods such as photochemical, electrochemical, and chemical reduction [14–16], which all use highly hazardous and expensive reagents and reductants [17]. Therefore, interest in green chemistry has been increasing, especially in recent years. According to Anastas and Warner [18], green chemistry includes principles that prevent or reduce the use of materials that threaten the environment and human health during the production and use of chemical products. Joining green chemistry principles with nanotechnology has been shown by Schmidt to be key to a sustainable society in the 21st century [19]. Green chemistry approaches have been used for synthesis of AgNPs with eco-friendly, safe, easy, and nontoxic methods. For this purpose, natural resources such as plants [20], yeast [21], and bacteria [22] are used in synthesis of AgNPs. In particular, plants do not need to be combined with any other agents in obtaining metal nanoparticles, because the bioactive molecules they contain can act as capping agents [23]. Therefore, researchers continue to work in this area, and interest in it has been increasing in recent years.

*Onosma sericeum* Willd. is a member of the family Boraginaceae and has been known for its wound healing properties in the Adıyaman region of Turkey for many years. The family Boraginaceae, which is dispersed in the tropical, subtropical, and temperate regions of the world, is represented by 100 genera and 2000 species [24]. Phytochemical analyses indicate that the family Boraginaceae, rich in naphthoquinone, includes alkannin, shikonin, and their derivatives [25]. However, we have found no studies in which this plant has been used in silver nanoparticle synthesis. Here, we report a novel and green method for synthesis of AgNPs using an extract of the root of *Onosma sericeum* Willd. as a reducing and stabilizing agent for the controllable synthesis of particles of different sizes at room temperature.

Our first objective was to develop a simple, economical, and eco-friendly method for synthesis of AgNPs using the root of *Onosma sericeum* Willd.. We wanted to develop a method that would not require any other organic solvent or stabilizing agent. We chose to optimize different parameters which can affect AgNP particle size, including temperature, pH, metal salt concentration, and root extract volume. Another aim was to demonstrate the biological and catalytical activity of the AgNPs. The cytotoxic effects of AgNPs of different particle sizes were investigated on the MCF-7 breast cancer cell line. The efficacy of the AgNPs as a catalyst used to reduce 2-nitrobenzenamine was also investigated. In addition, the antibacterial activity of AgNPs against a range of Gram-positive and Gram-negative bacteria was tested. When studies in the literature were analyzed, it was found that no study had been conducted on the synthesis of AgNPs using the root of *Onosma sericeum* Willd.; thus, the present study is the first of its kind.

## 2. Materials and methods

### 2.1. Chemicals and reagents

Analytical grade silver nitrate (AgNO3, 99%), 2-nitrobenzenamine (C6H6N2O2, 98%), RPMI 1640 medium, and MTT (3-(4,5-dimethylthiazol-2-yl)-2,5-diphenyltetrazolium bromide, 98%) were purchased from Sigma-Aldrich Corp. (St. Louis, MO, USA). Sodium tetrahydroborate (NaBH4, 98%) and dimethyl sulfoxide (DMSO, 99%) were purchased from Merck & Co., Inc. (Kenilworth, NJ, USA).

### 2.2. Preparation of extract

*Onosma sericeum* Willd. was collected from the Adıyaman-Besni region in Turkey (37°41ʹ35ʺN, 37°51ʹ33ʺE) from April to July 2018. The collected plant samples were first washed with tap water and then washed twice with distilled water to free them of dust and contamination. The plants were left to dry in a clean and dry place. For the extraction process, 200 mL distilled water was added to 20 g of roots broken into small pieces. This was subjected to maceration for 6 h at a temperature not exceeding 90 °C in a shaking water bath. The extract was then filtered and kept in a refrigerator during the rest of the operating time. Attention was given to preparing and using the extract while it was as fresh as possible.

### 2.3. Synthesis of silver nanoparticles

**Figure 1:**
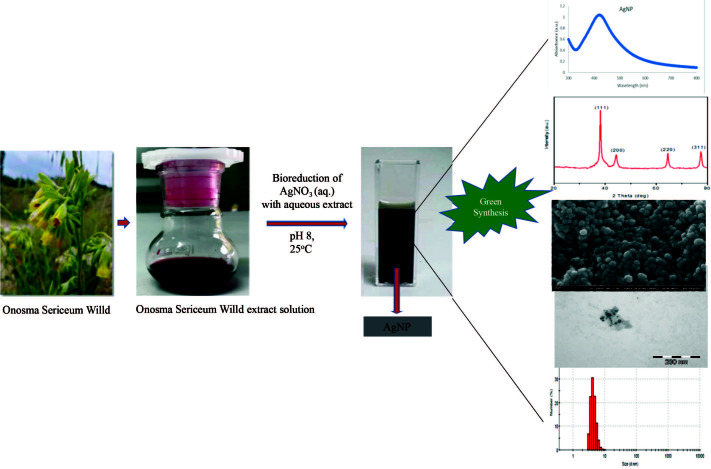
Schematic showing the synthesis procedure for AgNPs.

The extract (20 g/200 mL) was added dropwise into silver nitrate solutions which were prepared at different concentrations (1, 10, and 100 mM), and mixed with a magnetic stirrer. Silver nitrate and extract were used at a ratio of 5:1 (v/v). Within the first 10 min, the silver nanoparticle synthesis manifested itself in a brown-black color. As seen in Figure 1, these brown-black silver nanoparticles were separated through centrifugation and washed 3 times with deionized water in order to remove the plant extract residues. The processes performed for synthesis optimization were carried out at different temperatures and pH values.

### 2.4. Characterization of the synthesized AgNPs

#### 2.4.1. UV-visible and FTIR

The optical properties of the synthesized AgNPs were scanned between the range of 300–800 nm with a Shimadzu UV-1800 spectrophotometer set to a resolution of 1 nm (Shimadzu Corp., Kyoto, Japan). For this purpose, we put 200 µL AgNPs into a quartz cell and brought the sample up to a volume of 2 mL by adding deionized water. Infrared spectra of the extract and silver nanoparticles were obtained using a Jasco FTIR-6700 spectrophotometer in the wave number range 400–4000 cm−1 (Jasco, Inc., Easton, MD, USA).

#### 2.4.2. FESEM-EDAX and TEM

The structural morphologies of AgNPs were obtained using a Zeiss/Supra 55 field emission scanning electron microscope (FESEM) (Carl Zeiss Microscopy GmbH, Oberkochen, Germany). A high vacuum sputter platinum coating device was used for sample preparation. Elemental analysis of the silver nanoparticles was performed using an energy dispersive X-ray detector (EDAX). A transmission electron microscope (TEM) was used (JEOL JEM-1011; JEOL Ltd., Tokyo, Japan) to determine the size and shape of the synthesized AgNPs. TEM images were taken by drop-coating AgNPs on carbon-coated copper grids after sonication for 20 min.

#### 2.4.3. Particle size and zeta potential

The size distribution and zeta potential measurements of AgNPs were determined using a Zetasizer Nano-ZS (Malvern Instruments Ltd, Malvern, UK). Before size distribution and zeta potential measurements, AgNPs were suspended in deionized water at a concentration of 1 mg/10 mL, and then sonicated using a sonicator at room temperature for 20 min at 40 W to form a homogeneous suspension.

#### 2.4.4. XRD

The crystalline structure of the nanoparticles was determined with a high-resolution Rigaku SmartLab X-ray diffractometer (XRD) (Rigaku Corp., Tokyo, Japan) using Cu(Kα) radiation (wavelength: 1.54 Å) operating at 40 Kv and 40 mA at room temperature.

### 2.5. Catalytic properties

One of the important aspects of AgNPs is their catalytic effect. For the purpose of observing this effect, a combination of 10 mL of water, 1 mL of 1 mM 2-nitrobenzenamine, and 1 mL of 1 mM NaBH4 was added to a tube and mixed. Then, 1 mL of 0.01% silver nanoparticle solution was added to this mixture. Samples were taken from the obtained mixture at intervals of 1 min, measurements were made in the range of 200­–600 nm with a UV-Vis spectrophotometer, and reaction monitoring was performed. To confirm the catalytic properties of the synthesized AgNPs, this process was monitored in the absence of AgNPs. The control solution contained only NaBH4, 2-nitrobenzenamine, and water.

### 2.6. Cytotoxic activity

The MTT assay described by Khorrami et al. [26] was modified in this study. MCF-7 cells were seeded in a 96-well plate; 104 cells were placed in each well. At the end of 24 h, the silver nanoparticles were applied to the cells in final concentrations of 12.5, 25, 50, and 75 µg/mL in fresh RPMI 1640. Ten repetitions (n = 10) were performed for each concentration. After 24 h, the silver nanoparticles added to the cells were pulled from the wells and MTT solution (5 mg/mL) was added to the cells. The cells were incubated with 5% CO2 at 37 °C. After 5 h incubation, the medium in the cells was taken out, and 200 µL DMSO was added to each well. The plate was read in an ELISA reader set to 570 nm wavelength. The same steps were repeated during the following 48 h. We calculated cell viability at 24 and 48 h after application of silver nanoparticles according to Equation 1 below. IC50 values were then calculated by plotting the percentage values (%) of cell viability against logarithmic concentration.

Relative cell viability (%) = OD570 of the treated sample/OD570 of the control (Eq. 1).

### 2.7. Antibacterial activity

The in vitro antibacterial activities of the AgNPs were investigated against *Staphylococcus aureus* (ATCC 25925), *Escherichia coli* (ATCC 25923), *Acinetobacter baumannii* (ATCC 02026), *Bacillus subtilis (* ATCC 6633), and *Aeromonas hydrophila* (ATCC 95080) using the resazurin microtiter assay (REMA) method [27]. Ampicillin was used as the standard drug. The minimum inhibition concentration (MIC) value was determined.

## 3. Results and discussion

**Figure 2:**
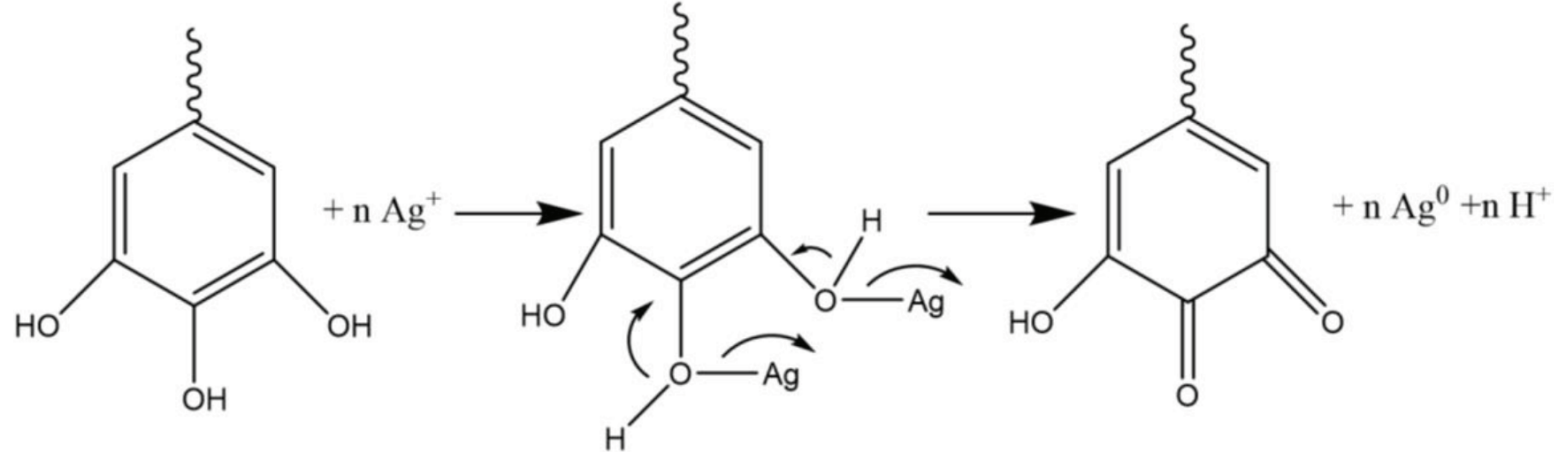
Possible mechanism of biosynthesis of AgNPs.

The genus *Onosma* is known to contain many metabolites such as aliphatic ketones, naphthazarins, alkaloids, flavones, naphthoquinones, and phenolic compounds [28]. These bioactive species play an active role in the reducing and stabilizing functions of AgNPs. The phenolic compounds in particular possess hydroxyl groups which are capable of nanoparticle generation. As shown in Figure 2, the nanoparticle formation mechanism is predicted to be due to tautomeric transformations from the enol-form to keto-form [29].

### 3.1. UV-visible and FTIR analysis

Silver nanoparticle synthesis was performed at silver nitrate concentrations of 1, 10, and 100 mM. Each synthesis was repeated under low (25 °C) and high temperatures (85 °C), and under acidic (pH 2) and basic (pH 8) conditions. The formation of silver nanoparticles was understood when a black/brown color appeared. A surface plasmon resonance (SPR) peak due to the collective oscillation of the free conduction band electrons in the visible region is characteristic and confirms the formation of AgNPs [30]. At all concentrations, it was observed that the characteristic plasmon band formed at 400–500 nm from the solutions of AgNPs. It was observed that the bandwidth and its wavelength varied depending on the silver nanoparticles’ size distribution, average diameter size, and pH of the medium [31]. For example, AgNPs with larger particle sizes were synthesized in all studies performed in the acidic medium at all concentrations. The characteristic plasmon bands of these nanostructures reached higher wavelengths (>450 nm, red shift), and wider peaks were obtained. However, AgNPs with smaller particle sizes were synthesized in all studies performed in the basic medium. The characteristic plasmon bands of these nanostructures reached lower wavelengths (400–450 nm, blue shift), and sharper peaks were obtained [32].

**Figure 3:**
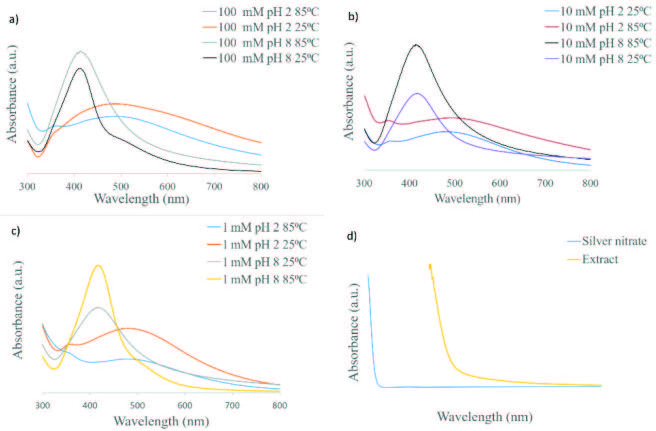
UV-Vis spectra of silver nanoparticles a) 100 mM, b) 10 mM, c) 1 mM, d) Silver nitrate (aq.) and extract of Onosma sericeum Willd.

Reaction pH in the biosynthesis of AgNPs is very important because of the ionization of the phenolic functional groups present in the *Onosma sericeum* Willd. extract, which is responsible for reduction and capping. At a lower pH, aggregation occurs more than nucleation, which occurs preferentially at a higher pH [33]. Nanoparticle aggregation seems to outcompete the nucleation process at pH 2, whereas a large number of nuclei form at pH 8. At pH 8, instead of aggregation, formation of nuclei led to the synthesis of higher numbers of nanoparticles with smaller diameters. A blue shift in the absorption pattern confirmed the formation of relatively smaller nanoparticles (Figure 3). Additionally, changes in pH may affect the capping and stabilizing properties of AgNPs [34]. We also observed that the intensity of the peaks increased with increasing silver nitrate concentration, suggesting that the number of AgNPs rises. Similar observations were reported previously by Samari et al. [35]. Plasmon bands of AgNPs obtained at 25 °C and 85 °C under acidic and basic conditions for 1, 10, and 100 mM are shown in Figures 3a–3c. Figure 3d shows the UV-Vis spectra of silver nitrate (aq.) and plant extract.

**Figure 4:**
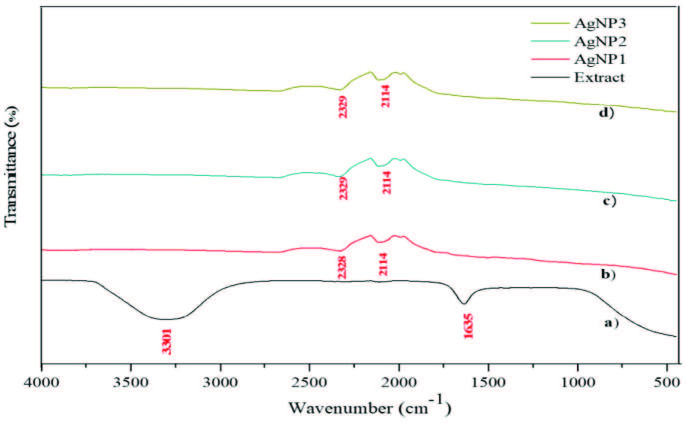
FTIR spectra of extract, AgNP1 (1–10 nm), AgNP2 (10–30 nm), and AgNP3 (30–50 nm).

The presence of biomolecules in the root extract of *Onosma sericeum* Willd., which is thought to play an active role in reducing silver nitrate to AgNPs, was confirmed by the FTIR analysis. The existence of a peak at 3301 cm–1 results from the –OH stretching of alcohols or phenols or from bending/stretching of hydrogen-bonded phenolic species in the extract. The bands seen in the FTIR spectra of the root extract in the range of approximately 1600–1650 cm–1 were assigned for stretch/bending vibrations [36]. The root extract, which is the leading source used in AgNP synthesis, is rich in phenolic and flavonoid species, and its antioxidant properties have been demonstrated in our previous study [37]. These phenolic structures, which are known as strong reducing agents [38], were confirmed by the FTIR spectra in which Ag1+ was reduced to Ag0. Figure 4a shows FTIR spectra of *Onosma sericeum* Willd. extract.

As shown in Figures 4b–4d, the bands within the 2100–2250 cm–1 range might be due to -C≡C or -C≡N. The presence of weak peaks indicated that the AgNPs might be capped by functional groups in *Onosma sericeum* Willd.

### 3.2. FESEM, TEM images, and particle size distributions

When the UV-Vis spectra were taken into consideration, the basic medium was chosen for the synthesis of AgNPs, since smaller nanoparticles were obtained in the basic medium than in the acidic medium. Although silver nanoparticles can be synthesized both at a low temperature (25 °C) and a high temperature (85 °C) in basic medium, it was seen that temperature directly affected the particle size of these metal nanoparticles [39]. It was found that these nanostructures were more prone to agglomeration, as more than one nucleus formed in the reduction of Ag1+ to Ag0 at the same time at high temperature. Thus, AgNPs having larger particle diameters were synthesized at high temperature. Silver nanoparticles with an average particle size greater than 100 nanometers were obtained (Supporting data 1). These silver nanoparticles had higher particle diameters due to the increase in their ability to agglomerate at high temperature. Therefore, it was decided that the most suitable condition for silver nanoparticle synthesis was basic pH and low temperature (pH 8, 25 °C).

**Figure 5:**
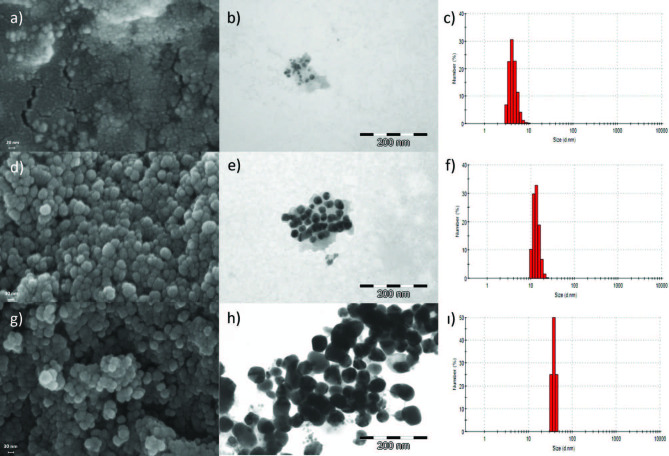
(a), (b), and (c) FESEM, TEM images, and particle size distributions of AgNP1 (1–10 nm), (d), (e) and (f) FESEM, TEM images and particle size distributions of AgNP2 (10–30 nm), (g), (h) and (i) FESEM, TEM images and particle size distributions of AgNP3 (30–50 nm) (25 °C, pH 8).

Three different silver nitrate concentrations were selected for comparison for synthesis of silver nanoparticles. These concentrations were 1, 10, and 100 mM. Our abbreviations for silver nanoparticles synthesized with 1, 10, and 100 mM silver nitrate are AgNP1, AgNP2, and AgNP3, respectively. FESEM, TEM images, and particle size distributions of silver nanoparticles AgNP1, AgNP2, and AgNP3 prepared at the ratio of 5:1 (v/v) with 20 g/200 mL plant extract at low temperature (25 °C) and under basic conditions (pH 8) are as indicated in Figure 5. According to the FESEM and TEM images, the AgNP1, AgNP2, and AgNP3 silver nanoparticles formed under the above conditions were spherical, and their particle sizes were between 1–10 nm, 10–30 nm, and 30–50 nm, respectively. When the concentration of silver nitrate was increased, the average diameter size increased. These results are in agreement with recent studies of biosynthesis of silver nanoparticles [31]. These results can be explained by the fact that bioactive compounds present in plant extract cause more nuclei formation in reducing silver nitrate, and these nuclei are easier to agglomerate. TEM images also showed that the especially small AgNPs were coated with an organic layer which acts as a capping agent, in agreement with results from a previous study [8].

### 3.3. EDAX analysis and zeta potential measurements

**Figure 6:**
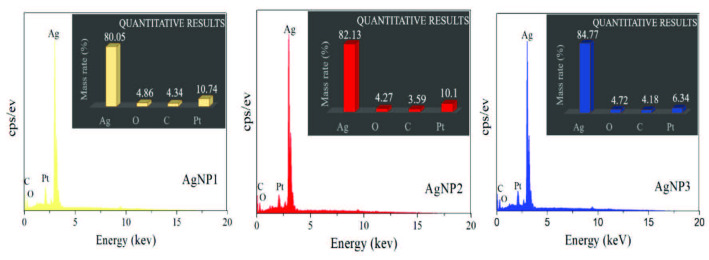
EDAX spectra of AgNP1 (1–10 nm), AgNP2 (10–30 nm), and AgNP3 (30–50 nm).

The elemental composition of the synthesized AgNPs was determined with an FESEM equipped with an EDAX detector. As shown in Figure 6, a strong signal at ∼3 keV indicated the presence of metallic silver. This result is consistent with those found in the literature [27]. The results showed that Ag was the main element in all AgNPs. The 2 elements that were present in small traces in comparison with Ag were carbon (C) and oxygen (O), which were thought to have derived from the root extracts. Platinum (Pt) was also found in small traces, and was thought to have come from the coating material used in the preparation of the samples. The relative Ag amounts of the 3 AgNPs were 84.77%, 82.13%, and 80.05% for AgNP3, AgNP2, and AgNP1, respectively.

It is known that zeta potential is an important parameter related to the stability of nanoparticles. The negative value of the zeta potential indicates that the AgNPs are covered with negatively-charged biomolecules, and that electrostatic interaction between these nanoparticles results in the long-term stability of metal nanoparticles by preventing possible aggregation [40].

In our study, the biomolecules existing in the *Onosma sericeum* Willd. plant and used as reducing and stabilizing agents in silver nanoparticle synthesis are thought to be responsible for electrostatic interaction between species.

**Figure 7:**
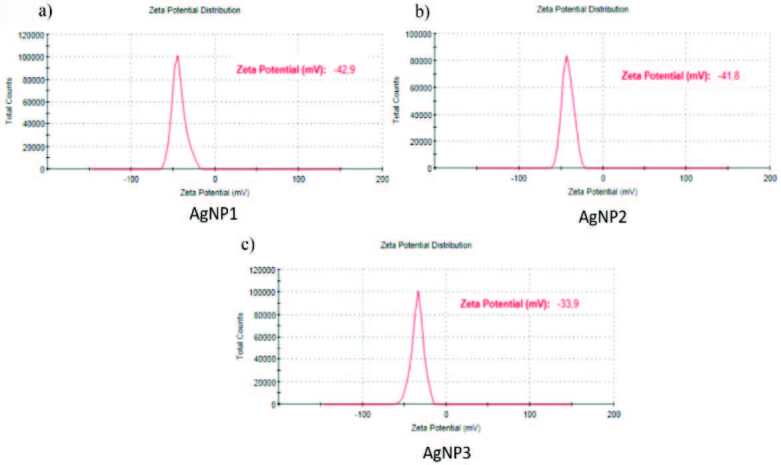
Zeta potential of a) AgNP1 (1–10 nm), b) AgNP2 (10–30 nm), c) AgNP3 (30–50 nm).

Zeta potential values (ζ-Pot, mV) at 25 °C for AgNP1, AgNP2, and AgNP3 were measured and determined to be –42.9 ± 3.67, *–* 41.8 ± 2.33, and –33.9 ± 1.55, respectively (Figure 7).

As can be seen from these results, AgNP1 (1–10 nm) and AgNP2 (10–30 nm), having small particle sizes, have higher zeta potentials, so they are more stable than other silver nanoparticles. This is because AgNP1 and AgNP2 synthesis was studied using a lower concentration of silver nitrate than other nanoparticles. Therefore, the added plant extracts were more effective in reducing silver ions. This also shows that the nanoparticle surface was covered more with negatively-charged biomolecules.

### 3.4. XRD analysis

**Figure 8:**
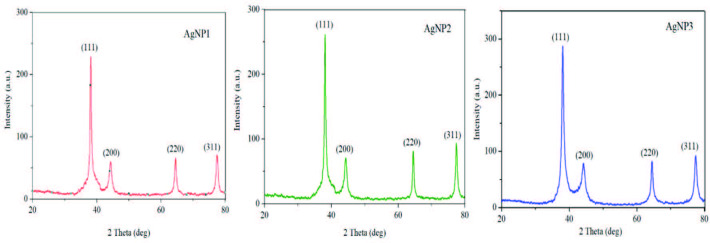
XRD spectra of AgNP1 (1–10 nm), AgNP2 (10–30 nm), and AgNP3 (30–50 nm).

An X-ray powder diffractometry technique was used to determine the crystalline structures of silver nanoparticles obtained by biosynthesis. The XRD patterns obtained for the 3 selected concentrations matched with the database (file no: 04-0783) of the Joint Committee on Powder Diffraction Standards (JCPDS). The XRD spectra obtained for AgNP1, AgNP2, and AgNP3 are shown in Figure 8. As a result of the obtained diffraction pattern, peaks corresponding to crystalline structures (111), (200), (220), and (311) were seen for elemental silver. This shows that the structure is fcc (face-centered cubic) metallic silver. These results are compatible with previous studies [41,42].

Another important parameter in the synthesis of AgNPs is the optimization of the amount of plant extract. The synthesis of AgNPs was repeated by taking 1, 5, and 10 mL of the plant extract. When the volume of extract was 1 mL, silver nanoparticle synthesis took longer (>2 h), and metal nanoparticles with a larger particle size (>100 nm) were obtained (Supporting data 2). This is due to the fact that the amount of polyphenol in the plant extract, which plays a role in the reduction, is lower [43]. As a result of this, it is possible to obtain metal nanoparticles with larger particle diameters by allowing the silver nuclei to come together more easily. When 5 mL of extract volume was used, silver nanoparticle synthesis occurred within the first 10 min, and no increase in particle size was observed. In the case of using 10 mL extract and 5 mL extract, there was no significant difference in either reaction time or particle size, so we preferred to use 5 mL as extract volume.

### 3.5. Catalytic activity

**Figure 9:**
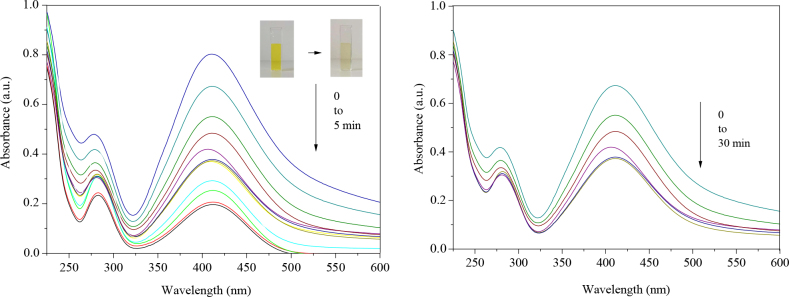
Reduction of 2-nitrobenzenamine a) with AgNP1 (1–10 nm) catalyst, b) absence of AgNP1 catalyst as the control samples.

It is known that even a very low concentration of 2-nitrobenzenamine is harmful for water ecosystems and human health in terms of its potential carcinogenic and mutagenic effects [44]. This chemical is included in the hazardous waste and toxic pollutant class by the U.S. Environmental Protection Agency [45]. Therefore, its removal is important for green chemistry. It is also known that the catalytic activity of metal nanoparticles is related to the particle size of these nanostructures [46]. Thus, we investigated whether AgNP1, AgNP2, and AgNP3, which were obtained from our study and had different particle sizes, had the potential to catalytically reduce the known toxicity of 2-nitrobenzenamine. It was determined that the absorbance intensity at 420 nm characteristic of 2-nitrobenzenamine changed very slowly without the addition of silver nanoparticles. However, when we read absorbances periodically after adding 0.01% AgNP solution to the same mixture, greater changes were observed. The most effective results were obtained when AgNP1 (1­–10 nm) was used. Within the first 5 min, the characteristic yellow color of 2-nitrobenzenamine and the absorbance intensity decreased noticeably (Figure 9a). However, when AgNPs were not added as a catalyst, the color change of 2-nitrobenzenamine took 30 min (Figure 9b).

**Figure 10:**
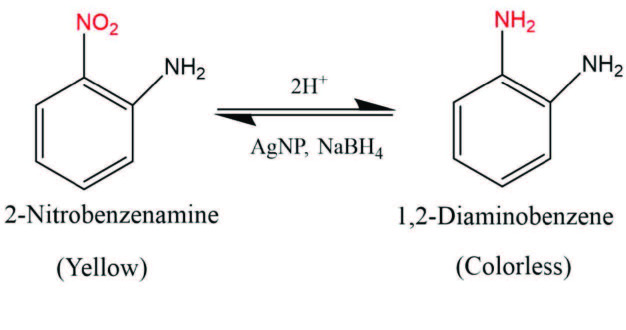
Catalytic reduction of 2-nitrobenzenamine to 1,2-diaminobenzene with AgNP catalyst.

Although the literature shows that silver nanoparticles have a catalytic effect in reducing 2-nitrobenzenamine to 1,2-diaminobenzene, the reduction time is 5 times faster in this study than in other studies [47,48]. Although the reduction of nitroanilines in the presence of NaBH4 seems thermodynamically possible, in the absence of a catalyst it is not kinetically very easy due to the large energy barrier between the electron donor and the electron acceptor species. AgNPs overcome the energy barrier and act as electron donors and acceptors simultaneously for the reduction of nitroanilines [49]. AgNPs play an important role in the transfer of electrons from NaBH4 to 2-nitrobenzenamine (Figure 10).

### 3.6. Cytotoxic activity

**Figure 11:**
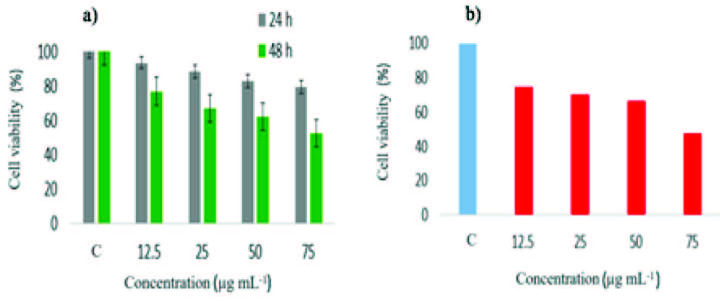
a) Cell viability of MCF-7 after 24 h and 48 h incubation with different concentrations of AgNP2 (10–30 nm), b) cell viability of MCF-7 after 48 h incubation with different concentrations of AgNP1 (1–10 nm).

Antibacterial effects of silver nanoparticles have been shown in many studies [50,51]. However, studies on their cytotoxic effects are limited. In our study, the cytotoxic effects of the 3 different synthesized and characterized AgNPs, which have different particle sizes, were studied. For this experiment, MCF-7 cells were used in an MTT test, a colorimetric method. Different concentrations (12.5 µg/mL, 25 µg/mL, 50 µg/mL, and 75 µg/mL) of AgNP1, AgNP2, and AgNP3 were added to MCF-7 cells. The percentage values (%) of relative cell viability after 24 h and 48 h were determined using Equation 1. In addition, IC50 values were calculated. AgNP3 showed no cytotoxic effect on MCF-7 cells at any concentration or time period studied. However, AgNP2 (10–30 nm) showed a cytotoxic effect on cancer cells at both 24 h and 48 h (Figure 11a). It was concluded that these nanoparticles reduced the percentage (%) of MCF-7 cancer cells’ viabilities depending on increasing concentration and time and were therefore cytotoxic. For AgNP2, the calculated percentage rates (%) of living cells at the end of 24 h were determined as 93.525% ± 5.05, 88.465% ± 7.52, 82.962% ± 5.93, and 79.315% ± 8.32 for the concentrations of 12.5 µg/mL, 25 µg/mL, 50 µg/mL, and 75 µg/mL, respectively. The calculated percentage rates (%) of living cells at the end of 48 h were 77.03% ± 12.21, 66.96% ± 10.35, 62.29% ± 8.41, and 52.49% ± 13.2 for the concentrations of 12.5 µg/mL, 25 µg/mL, 50 µg/mL, and 75 µg/mL, respectively.

The IC50 value of AgNP2 was 169.77 µg/mL at 24 h and 81.708 µg/mL at 48 h. AgNP1 (1–10 nm) showed a cytotoxic effect on cancer cells depending on concentration but only at 48 h (Figure 11b). For AgNP1, the calculated percentage rates (%) of living cells at the end of 48 h were 74.67% ± 7.93, 70.48% ± 8.19, 66.67% ± 7.13, and 47.38% ± 12.63 for the concentrations of 12.5 µg/mL, 25 µg/mL, 50 µg/mL, and 75 µg/mL, respectively. The IC50 value of AgNP1 was 76.63 µg/mL at 48 h. As can be seen from the results, particle size had a significant effect on the cytotoxic effects of silver nanoparticles, and the two most cytotoxic silver nanoparticles were below approximately 20 nm. Another finding from the study is that the cytotoxic effect increases as concentration and time increase. However, it will be necessary to perform studies on different cell lines to determine whether the AgNPs have cytotoxic effects on normal cells as on cancer cells, or if this occurs only at much higher concentrations. Park et al. [52] indicated that AgNPs which had a smaller particle diameter showed higher cytotoxic effects, and this finding supports our study. The mechanism of cytotoxic action of silver nanoparticles on cells may involve the induction of oxidative stress by way of the formation of free radicals and the production of reactive oxygen species (ROS).

### 3.7. Antibacterial activity

Ag is known to have antibacterial properties, and for this reason it has been used in many medical applications against various pathogens over time. AgNPs are able to interact with bacterial cell membranes due to their large surface area. In recent years, studies carried out on these nanostructures have shown that that their potential for antibacterial activity is closely related to membrane damage and DNA toxicity [53]. In studies in the literature, AgNPs were found to be effective against bacteria such as *Pseudomonas aeruginosa* , *Proteus mirabilis* , *Escherichia coli* , *Shigella flexneri* , *Shigella sonnei* , and *Klebsiella* [54,55].

The present study investigated whether 3 nanoparticles with different particle sizes were effective against *Staphylococcus aureus* , *Escherichia coli* , *Acinetobacter baumannii* , *Bacillus subtilis* , and *Aeromonas hydrophila* . The MIC values of AgNP2 (10–30 nm) and AgNP3 (30–50 nm) were in the range of 250–500 μg/mL for *Staphylococcus aureus* , *Escherichia coli* , *Acinetobacter baumannii* , *Bacillus subtilis* , and *Aeromonas hydrophila* . AgNP1 (1–10 nm) showed antibacterial activity at 125 μg/mL for *Acinetobacter baumannii* (ATCC 02026). This value is the same as the MIC value of ampicillin used as the standard drug (Table 1). This result is of great importance for the dangerous pathogen *Acinetobacter baumannii* , which causes hospital-acquired infections and has multiple drug resistance.

**Table 1:** The MIC values (µg/mL) of AgNPs and ampicillin against bacteria.

AgNPs	Staphylococcus aureus(ATCC 25925)	Escherichia coli(ATCC25923)	Acinetobacter baumannii(ATCC 02026)	Bacillus subtilis (ATCC 6633)	Aeromonas hydrophila (ATCC 95080)
*AgNP3	500	250	500	500	500
*AgNP2	250	250	250	250	250
*AgNP1	250	125	125	125	250
Ampicillin	31.25	15.62	125	0.90	31.25

*AgNP1 (1–10 nm), AgNP2 (10–30 nm) and AgNP3 (30–50 nm).

## 4. Conclusion

In this study, AgNP synthesis was carried out using *Onosma sericeum* Willd. aqueous extract for the first time. We used a simple and economical method and an approach that favors green chemistry. We concluded that the metal salt concentration, root extract volume, temperature, and pH were determinants of the particle size, which in turn affected the chemical behavior of the AgNPs. Therefore, the catalytic properties and biological activity of the 3 different AgNPs were evaluated by optimizing these parameters. The AgNPs with a particle size under 10 nm showed better performance in reducing 2-nitrobenzenamine in a very short time. Additionally, these nanoparticles were found to be cytotoxic against the MCF-7 breast cancer cell line depending on dosage and time. One of the most important results of the present study was that the synthesized AgNPs were effective against the *Acinetobacter baumannii* bacteria, a known opportunistic pathogen associated with nosocomial infections.

The results from the present study and from other reported studies of synthesis of silver nanoparticles with plant extracts and their applications are compared in Table 2. As can be seen in Table 2, it is possible to find studies on silver nanoparticle synthesis using plant extracts in the literature. However, no silver nanoparticle synthesis has been previously studied with *Onosma sericeum* Willd. More importantly, in this study, the catalytic, cytotoxic, and antimicrobial efficacy of silver nanoparticles with particle sizes under 10 nm were evaluated together. It can be concluded that AgNPs, synthesized from the root extract of *Onosma sericeum* Willd., and whose particle size can be determined by optimizing different parameters, have the potential to play an active role in future drug development processes, as well as biomedical and nanobiotechnology applications.

**Table 2:** Comparison of the results of the present study and other studies on synthesis of AgNPs.

Plant	Size and shape	Applications	Reference
Amaranthus retroflexusVerbascum thapsus	10–32 nm, spherical8.4–48.7 nm, spherical	Antifungal activityPhotodegradation of nitrobenzene	[56][57]
Coffea arabica seed	20–30 nm, spherical	Antibacterial activity on E. coli and S. aureus	[58]
Pedalium murexGelsemium sempervirens	20–50 nm, spherical 112 nm, spherical	Antibacterial activity against E. coli, K. pneumonia, M. flavus,P. aeruginosa, B. subtilis, B. pumilus, and S. aureus. Cytotoxicity in A375 cells	[59][60]
Onosma sericeum Willd.	1–10 nm, spherical	Antibacterial activity against the Acinetobacter baumanniibacteria Showed higher catalytic effect in the reduction of 2nitrobenzenamine Cytotoxic effect on MCF-7 cells	This study

## Funding

This study was funded by the Mersin University Scientific Research Project Unit with the project no. 2019-1-AP2-3412.

Supplementary Materials
