# Supplementary material for: Biosynthesis of silver nanoparticles using Onosma sericeum Willd. and evaluation of their catalytic properties and antibacterial and cytotoxic activity
Source: Turk J Chem. 2020 Dec 16;44(6):1587–600. doi: 10.3906/kim-2007-1 (PMC7765767; doi:10.3906/kim-2007-1)
Supplement: Supplementary file 1 — Supplementary Materials [file turkjchem-44-1587-sup001.pdf]

Electronic Supplementary Information

Biosynthesis of silver nanoparticles using *Onosma sericeum Willd* and evaluating the catalytic properties and antibacterial and cytotoxic activity

Selda DOĞAN ÇALHAN[1, *], Mustafa GÜNDOĞAN[2]

[1] Department of Pharmaceutical Biotechnology, Faculty of Pharmacy, Mersin University, 33169 Mersin, Turkey

[2] Department of Pharmaceutical Technology, Faculty of Pharmacy, Mersin University, 33169 Mersin, Turkey

*Correspondence: seldadgn@gmail.com, seldadgn@mersin.edu.tr

ORCIDs:

Selda DOĞAN ÇALHAN: https://orcid.org/0000-0003-2589-8585

Mustafa GÜNDOĞAN: https://orcid.org/0000-0002-5063-6301

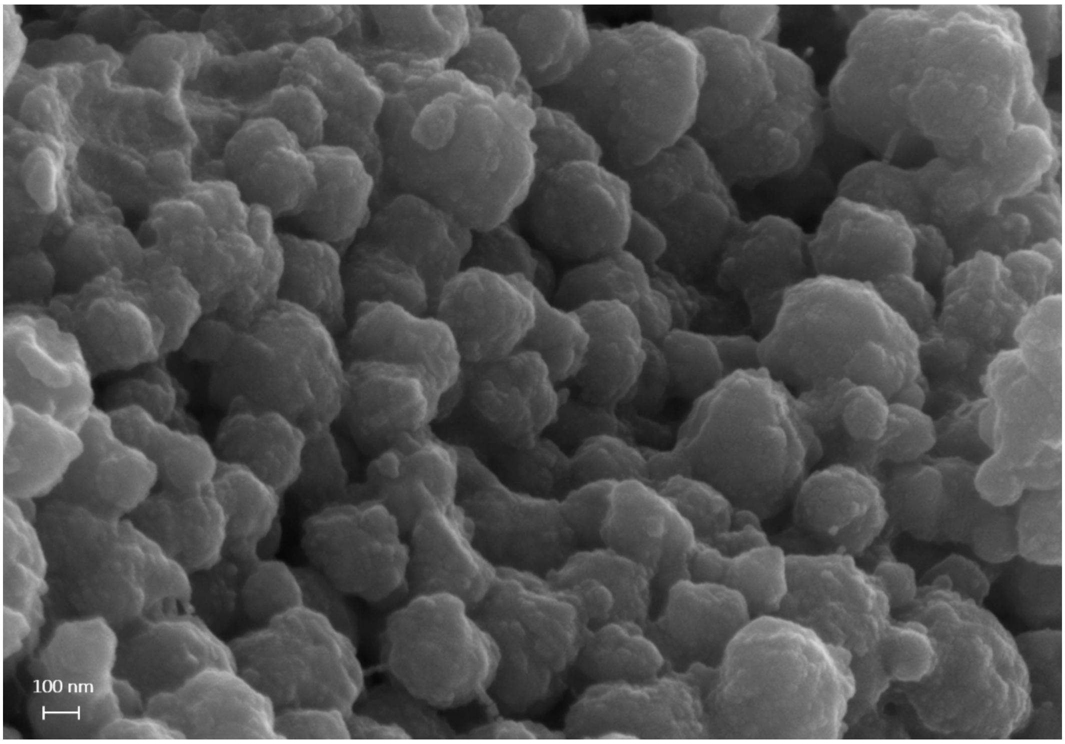

Supportin data 1. FESEM image of AgNP (85°C, pH 8).

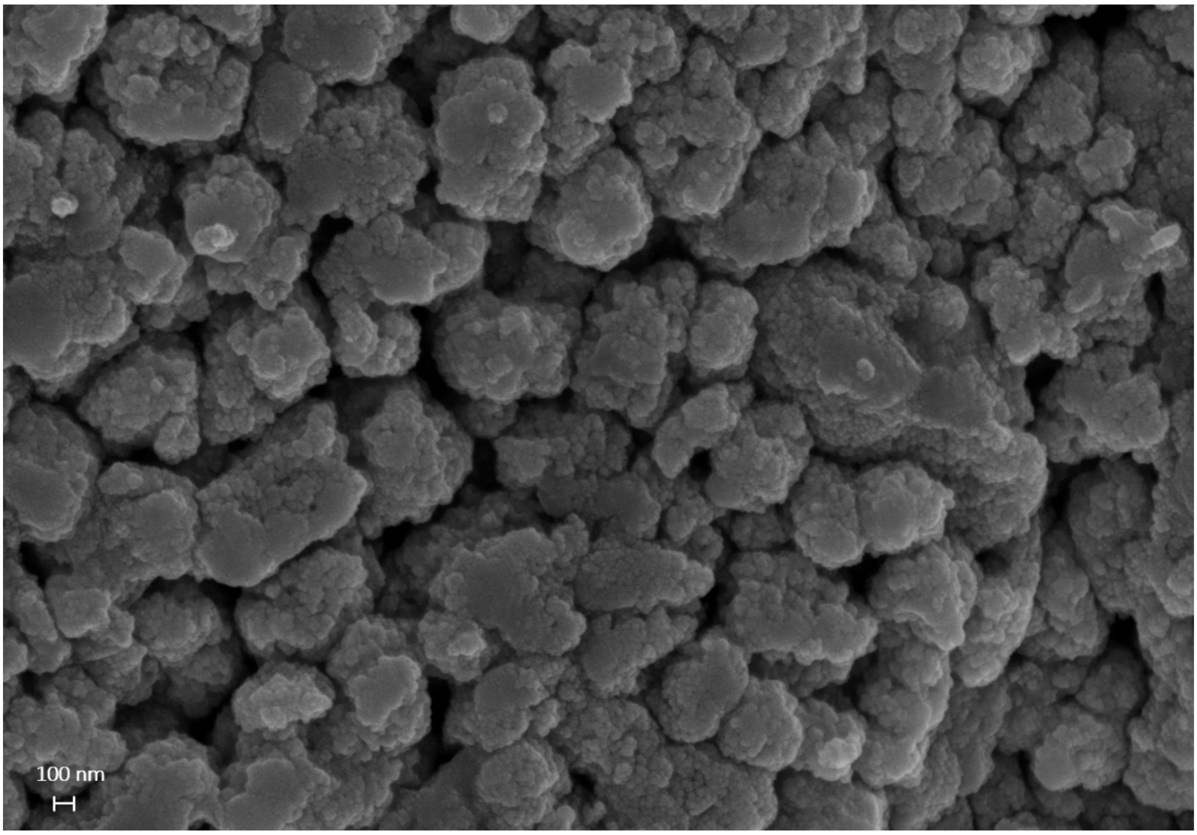

Supportin data 2. FESEM image of AgNP (volume extract 1 mL)
